# Restoration of Sick and Wounded to the Line

**Published:** 1918-12

**Authors:** 


**The Restoration of the Sick and Wounded to the Line.** Colonel BILLINGS, M. C.

I am very glad to have the opportunity to tell you something of the plans of the War Department, which have been approved by the General Staff and the Secretary of War.

First of all, of course, the sentiment which created these plans was that our soldiers in the line of duty should not be treated as the soldiers were in the Civil War. We should not consider them from a purely sentimental point of view because of the fact that conscription has been made law in America and, whether willing or not, has brought our men into the army. They therefore deserve recognition by the government and proper treatment.

Consequently a certain general formed a plan called the " Physical Reconstruction of Men ". It took a long time to educate the officials of the War Department as to what this plan was. Very little progress was made until this spring, and from then on until July it took constant personal effort on the part of the personnel of the division to get this plan approved by the War Department.

The plan, in principle, quotes several things :

1. The policy is that no soldier disabled in the line of duty, even though he is not able to return to duty, shall be discharged from the army until he shall have recovered or as nearly recovered as possible. So it makes no difference what the disability of an American soldier is; he will not be discharged until he is as nearly cured as conditions permit.

2. Continued treatment until physical and functional cure.

In applying these principles in the treatment of soldiers, the War Department has approved the plan of a certain general to utilize every modern means of medical and surgical treatment to that end, and to adopt in each hospital at home a physiotherapeutic department with special buildings, modern equipment, and a personnel, including women from civil life, for massage and other physical means of treatment.

Three types of buildings for physiotherapy have been adopted.

Curative work is recognized as one of the methods of treatment, both physical and mental. Here, great difficulty was experienced in convincing the staff that education is a means of treatment. When this was finally approved, permission was obtained to bring into every hospital educators from civil life, and to commission them in the Sanitary Corps. This plan was approved in the latter part of July and published on August 1st. It permits us to commission education officers in the Sanitary Corps, to create administrative education offices, and to have an education force in every hospital composed of general educators and technical educators. The necessary personnel is obtained from among disabled soldiers. The plan enables us to secure not only administrative education officers, but also instructors. The work was begun last March, though the plan had not yet been approved.

The instructors are chiefly disabled soldiers (90 o/o in 16 hospitals); they are either qualified in some trade, or are teachers in colleges, universities, high schools, etc. A telegrapher made a splendid teacher; he had a class of ten in one week.

In one hospital 20 trades are being taught. Workshops are attached to every general hospital and the trades taught are suitable to the different diseases of the men.

We have three types of workshops, and general educational buildings besides.

The head of this service, in the Surgeon General's office, is J. E. Russell who could not accept a commission because he was not physically fit for the army. · By his advice we have succeded in getting other educators. Captain... (Granger?) is the director for physiotherapy. Provision is made for playgrounds and gymnasiums as a part of physiotherapy.

Fred Welks, the pugilist, accepted a commission and is the physical sports director at one of the hospitals; he attends to the organization of football, baseball, etc.

The educational force includes civilian women, qualified as teachers in universities, high schools, art schools, etc. They are attached to the different hospitals. The members of the educational force are sent over here on requisition made by General Ireland; twenty women, ten of whom have specialized in physiotherapy and ten in hand crafts, are attached to every unit coming overseas, and are assigned to base hospitals where hand crafts and physiotherapy may be carried on.

This organization is as yet quite new in America, and therefore needs more and more publicity. To that end, the Surgeon General issues a little magazine called " Carry On " for the purpose of educating the public and the medical officers. Arrangements have been made with all of the moving picture companies of America, and with actors, so that moving pictures are formed and scenarios written. One of these was the reconstruction of a disabled man, beginning in France and finishing at home. Other arrangements have been made so that stories are written on the subject of physical reconstruction for the leading papers. We have got to reach the family of the man, and that is done through the Red Cross, which gets out literature and spreads it into the families of soldiers. We want to get this information to the soldier himself, both here and on the other side. One reason for my being ordered over here was that I might meet medical officers serving in this country and thus reach the men in the hospitals. Officers, nurses and soldiers are to be told of the privilege of a man who is no longer fit for military service, and of the opportunity to be trained for an occupation before he returns to civil life.

In a sense it is a development of the man. We do not use the name " Convalescent Camps " but that of " Development Battalions " to remove any idea of the man being an invalid, and to give no impression that it is a clinical thing. A man is assigned to duty in a development battalion whether he has broken down in the line of duty or has just come out of hospital. It is part of our duty to have advisory administrative power over that battalion, for obtaining the personnel and necessary specialists. The men are not considered as having any specific disease, but they are considered as men fit for some sort of graduated exercise, drill, etc. They are examined frequently by a medical officer, sent back to the base hospital if illness occurs, or classified and sent back to duty, discharged, or sent for long treatment of some disability.

May I ask that every man serving overseas learn something of the Surgeon General's plan of reconstruction. Make it known, tell it to the medical officers, to the nurses, to the men themselves.

We are not sending men back to civil life if they are fit for any military service; if they are not fit for the fighting line we train them so that they are fit for domestic service, or for special service over here to release combatant men in the rear. If they are not fit for any service whatever we get them into a hospital at home for physical reconstruction.

There is a board that has been charged by the government with the physical training of disabled soldiers, but it has no jurisdiction over a man until he is discharged from the army, and only over men who need training for a new job, and who choose to go in for this training. Our job is to treat the man, to educate him and to train him for other military service, or to make him of use in civil life as long as he remains a soldier, which is until he shall have been cured, or as nearly so as his disability permits.

